# Comparative Performance of Calcium Phosphate Grafts and Iliac Crest Autograft in Posterolateral Spinal Fusion in Rabbits

**DOI:** 10.1002/jsp2.70101

**Published:** 2025-07-30

**Authors:** William R. Walsh, Rema A. Oliver, Matthew H. Pelletier, Tian Wang, Chris Christou, Emma R. Walsh, Jonathan M. Page, Chase T. Davis, Gregory M. Williams

**Affiliations:** ^1^ Surgical and Orthopaedic Research Laboratories University of New South Wales, Prince of Wales Hospital Sydney Australia; ^2^ NuVasive Inc San Diego California USA; ^3^ Globus Medical Inc Audubon Pennsylvania USA

**Keywords:** animal model, biomaterials, bone graft substitute, calcium phosphate, iliac crest autograft, preclinical, spine fusion, synthetic bone graft

## Abstract

**Background:**

Calcium phosphate (CaP) biomaterials are widely used in surgical applications such as spinal fusion to substitute for or extend autogenous bone graft. Preclinical testing in standardized animal models is useful for evaluating the relative performance of materials differing in composition and structure, including a newer generation of submicron‐structured CaP (sCaP) with surface features uniformly smaller than 1 μm. The purpose of this study was to compare three clinically available CaP‐based materials and iliac crest autograft in the rabbit posterolateral fusion (PLF) model.

**Methods:**

A novel sCaP with bovine collagen type I (sCaP/Col I) and two clinically established materials, sCaP with alkylene oxide copolymer (sCaP/AOC) and microstructured CaP with bovine collagen type I (mCaP/Col I), were evaluated in a skeletally mature, single‐level, non‐instrumented, bilateral rabbit PLF model. Iliac crest autograft served as a control. Endpoints included radiographic, mechanical, and histological evaluation at postoperative 6, 9, and 12 weeks.

**Results:**

Fusion progressed with postoperative time with all grafts, and the CaP materials yielded fusion rates by micro‐CT and manual palpation similar to those of the autograft control at each time point. When tested as autograft extenders, sCaP/Col I and sCaP/AOC demonstrated equivalent results for all endpoints. When hydrated with bone marrow aspirate and used as bone graft substitutes, sCaP/Col I supported earlier fusion than mCaP/Col I with an increased radiographic fusion rate at 9 weeks (*p* = 0.032) and increased bone tissue content by histomorphometry at 12 weeks (*p* = 0.006). New bone was observed to form with all materials, and no adverse local biological reactions were seen.

**Conclusions:**

Differences in the composition and structure of clinically available CaP‐based materials influenced the achievement of spinal fusion in a standardized rabbit PLF model. These results may help guide the selection and use of materials in clinical applications and the future development of biomaterials with improved performance.

## Graphical abstract

Three clinically available calcium phosphate‐based bone grafting materials and iliac crest autograft were compared in a standardized model of posterolateral spinal fusion in rabbits. Graft materials were found to support a progression of fusion with postoperative time similar to autograft controls. Differences in the rate and quality of fusion, notably between submicron‐structured and microstructured calcium phosphate biomaterials, may help guide the selection and use of materials in clinical applications.

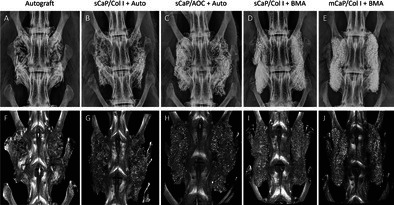

## 1 Introduction

The generation of bioceramics with an improved capacity for bone healing continues to be a promising solution for cost‐effective spinal fusion and bone void filling surgeries [1]. Optimizing the microstructure of calcium phosphate (CaP) ceramics has emerged as a leading engineering strategy to direct and enhance cellular responses towards these materials [2]. CaP with surface structures tailored within a narrow submicron range has been shown to be capable of improving bone formation in interspinous sites and even initiating bone formation in intramuscular sites lacking committed osteogenic cells in animal models [3, 4]. Recent mechanistic studies suggest that the immunologic reaction to CaP surface structures plays a key role in the formation of bone. Specifically, increased M2 polarization of macrophages in response to submicron‐structured CaP (sCaP) versus M1 polarization with microstructured CaP (mCaP) differentially stimulates bone and fibrous tissue formation, respectively [5, 6]. While many CaP‐based bone grafts currently available to surgeons have surface structures that fall outside of the submicron range, eliciting little or no bone formation upon intramuscular implantation, a bone graft putty (Attrax Putty, NuVasive Inc., CA, USA) incorporating sCaP in an alkylene oxide copolymer (AOC) binder is now clinically available [7, 8].

Recent clinical evidence supports the ability of the sCaP bone graft putty to perform as well as autogenous bone in spinal fusion applications. In a randomized controlled trial in instrumented posterolateral fusion (PLF), the rate of fusion as assessed by computed tomography (CT) with sCaP putty was noninferior to that of autograft at 1 year postoperative and continued to be comparable at 2 years [9, 10]. An additional randomized controlled trial of the sCaP putty in lateral lumbar interbody fusion (LLIF) also found the fusion rate at 1 year to be similar to that of iliac crest autograft [11]. These comparative trial results are reinforced by additional case series of the use of sCaP putty in cervical and lumbar spinal interbody fusion surgeries which demonstrate successful radiographic fusions and improved patient outcomes [12, 13, 14, 15].

With the increasing clinical adoption of sCaP graft materials, new formulations have been developed to address a wider range of surgical applications and clinician needs. Notably, the substitution of the sCaP putty's nonabsorbent AOC binder with a bovine collagen type I (Col I) matrix has produced a novel formulation (Attrax Scaffold, NuVasive) designed for compression and migration resistance as well as improved absorbency of biological fluids laden with cells and growth factors. However, the in vivo performance of this novel graft formulation has yet to be characterized in the literature.

Standardized in vivo models of bone healing and fusion have frequently been used to compare graft materials in the preclinical setting. Boden et al. developed a model of non‐instrumented, intertransverse process fusion in rabbits that closely replicates the human clinical procedure of intertransverse PLF without instrumentation in technique and outcomes [16]. In the decades following its development, the rabbit PLF model has become a preferred regulatory science tool by the United States FDA for the premarket evaluation of bone void fillers and has been widely used in the development of CaP‐based grafts and other biological materials [17, 18, 19, 20]. Importantly, well‐controlled studies using the rabbit PLF model can be helpful to assess the relative performance of graft materials in advance or in lieu of further clinical study.

This study evaluated the in vivo performance of a novel sCaP/Col I graft material as both a bone graft substitute and an autogenous bone graft extender in a standardized rabbit PLF model. This graft material was compared to a control of autologous iliac crest bone graft and two clinically established CaP‐based bone graft materials, including sCaP/AOC putty and a microstructured CaP/Col I graft (mCaP/Col I), to assess their relative fusion performance as influenced by differences in formulation, such as binder composition and CaP microstructure.

## 2 Materials and Methods

### 2.1 Test Articles and Characterization

Three bone grafting materials were procured for testing: sCaP/Col I (Attrax Scaffold, NuVasive), sCaP/AOC (Attrax Putty, NuVasive), and mCaP/Col I (Formagraft, NuVasive). Material composition was provided by the manufacturer through product labeling and supporting documentation (Table 1). Supplemental microstructural characterization was conducted on ~0.5 cc of each material by scanning electron microscopy (SEM) (FEI Apreo, Thermo Fisher Scientific Inc., MA, USA) and mercury intrusion porosimetry (Autopore IV/V, Micromeritics Instrument Corporation, GA, USA) [7, 8]. To assist with the characterization of the sCaP/AOC without the masking effect of the binder, the sCaP granules were first extracted by burning off the organic AOC binder at 800°C for 3 h. The other test articles were assessed with collagen binders intact.

**TABLE 1:** Graft material test article identification and composition.

Test article	Brand name (Manufacturer)	Ceramic composition	Ceramic granule size (mm)	Binder
sCaP/Col I	Attrax Scaffold (NuVasive)	> 90% β‐TCP; < 10% HA	0.5–1.0	Bovine Collagen Type I
sCaP/AOC	Attrax Putty (NuVasive)	> 90% β‐TCP; < 10% HA	0.5–1.0	Alkylene Oxide Copolymer
mCaP/Col I	Formagraft (NuVasive)	40% β‐TCP; 60% HA	0.5–1.0	Bovine Collagen Type I

Abbreviations: β‐TCP = beta tricalcium phosphate; AOC = alkylene oxide copolymer; CaP = submicron‐structured calcium phosphate; Col I = collagen type I; HA = hydroxyapatite; mCaP = microstructured calcium phosphate.

### 2.2 Animal Model and Experimental Design

With institutional ethics approval (UNSW Animal Ethics Committee, NSW, AU approval 14/110A), 90 skeletally mature female New Zealand White rabbits ( *Oryctolagus cuniculus* ) were selected to receive single‐level (L4–L5) non‐instrumented bilateral PLF as originally described by Boden et al. [16] Study subjects were assigned to five treatment groups and three postoperative durations of 6, 9, and 12 weeks (Table 2). Sample sizes were based on well‐accepted group sizes for comparative biomaterials evaluation in this model as previously published and were weighted towards the primary fusion endpoint at 12 weeks [18, 19]. Test articles were used either as bone graft extenders (i.e., combined 1:1 by volume with autologous iliac crest bone) or bone graft substitutes following hydration with autologous bone marrow aspirate (BMA) in a manner consistent with the applicable graft material indications for use.

**TABLE 2:** Experimental design summary.

Test group	Graft volume per side		Subjects (*n*)		
	Auto	CaP	6 weeks	9 weeks	12 weeks
Autograft	2 cc	—	5	5	8
sCaP/Col I + Auto	1 cc	1 cc	5	5	8
sCaP/AOC + Auto	1 cc	1 cc	5^a^	5	8
sCaP/Col I + BMA	—	2 cc	5	5	8
mCaP/Col I + BMA	—	2 cc	5	5	8

*Note:* Test groups were defined by their volumetric composition of autograft (Auto) and calcium phosphate (CaP)‐containing graft material mixed and implanted per side. Sample sizes indicate the number of animal subjects undergoing single‐level posterolateral fusion.

Abbreviation: BMA = bone marrow aspirate.

^a^ One subject was removed from analysis due to a health complication.

### 2.3 Surgical Methods and Animal Care

The standardized preoperative assessments, including confirmation of skeletal maturity by radiographic observation of growth plate closure, and the surgical operations performed by our institution for rabbit PLF subjects were performed as described in detail previously [17]. Additional study‐specific methods are as follows. Blood samples were processed for biochemistry and hematology analysis preoperatively and at euthanasia for each subject.

Iliac crest bone graft was harvested bilaterally from subjects in the autograft group and unilaterally from subjects in the sCaP/Col I with autograft and sCaP/AOC with autograft groups. The corticocancellous autograft bone was morselized with manual instruments into small pieces (< 5 mm) [3]. Per manufacturer's instructions for use, when used in combination with autogenous bone graft, sCaP/Col I was first hydrated with an equal volume of normal saline until fully saturated, while the non‐absorbent sCaP/AOC putty was used without any hydrating fluid. Both materials were then molded by hand and mixed with equal volumes (1 cc:1 cc) of autogenous iliac crest bone graft prior to implantation.

Bone marrow was aspirated from the proximal tibia of subjects in the sCaP/Col I with BMA and mCaP/Col I with BMA groups using a 25 mm, 18 g trocar needle. Per manufacturer's instructions for use, sCaP/Col I and mCaP/Col I were hydrated with an equal volume of BMA immediately after aspiration until materials were fully saturated prior to implantation.

Following surgical exposure and preparation of the spinal fusion sites, each subject received 2 cc of their group's designated graft material on each side of the spine overlying the intertransverse ligament and spanning the decorticated transverse processes of L4 and L5. Surgical wounds were closed and postoperative care was performed as previously described [17]. At postoperative durations of 6, 9, and 12 weeks, assigned subjects from each treatment group were humanely euthanized by cardiac injection of *pentobarbitone sodium* while under anesthesia, and spinal segments were excised.

### 2.4 Manual Palpation and Biomechanical Testing

Immediately following harvest, each operated spinal segment was assessed by the concurrence of two experienced and blinded investigators using manual palpation in flexion–extension and lateral bending as described previously [17, 18, 19, 20]. Adjacent, non‐operated segments were used as comparative unfused control segments. Fusion was defined as a rigid segment with no movement detected between vertebrae, whereas non‐fusion was defined by a nonrigid segment with movement.

Additionally, at the 12‐week postoperative time point only, non‐destructive robotic range of motion testing was used to quantify movement of the operated segments in flexion–extension (FE), lateral bending (LB), and axial rotation (AR). Spines were potted in custom molds with resin and tested using a Denso robot (simVITRO, Cleveland Clinic BioRobotics Lab, OH, USA) as described previously [18, 19]. Pure moments of 270 N·mm were applied at a rate of 33.3 N·mm/s to a maximum of 300 N·mm and held for 15 s. For each profile, 4.5 load–unload cycles were run, and the last three cycles were analyzed. A mean value at 270 N·mm was taken for each cycle and averaged. Data were compared to control data from non‐operated, skeletally mature female animal subjects (*n* = 5) previously established by the test facility.

### 2.5 Radiography and Micro‐CT

Radiographic assessments included imaging by Faxitron radiography (Faxitron Bioptics LLC, AZ, USA) with digital cassettes and by micro‐CT using an Inveon scanner (Siemens Medical Solutions USA Inc., PA, USA) per previously published methods [17, 18, 19]. Micro‐CT raw data were reconstructed in DICOM format at 53 μm resolution using the Siemens software. Micro‐CT images, including the full multiplanar image stacks along with 3D models, were assessed by the concurrence of two experienced and blinded investigators for bilateral fusion defined as a Lenke‐type grade A [21].

### 2.6 Histology and Histomorphometry

Spines were fixed for a minimum of 96 h in 10% formalin in 0.145 M phosphate buffered saline immediately following mechanical and radiographic endpoint testing. Fixed spines were bisected along the sagittal central axis. One side was randomly processed for decalcified paraffin histology while the other was processed for undecalcified polymethyl methacrylate (PMMA) histology per previously published methods [18, 19]. Spinal sides processed for paraffin histology were decalcified in 10% formic acid in phosphate buffered formalin for 3–4 days. The sides were then cut sagittally into four sections of ~3 mm thickness spanning the medial to lateral aspects of the fusion mass and processed into paraffin cassettes. Representative sections (~5 μm) across the fusion mass were cut from these paraffin blocks using a Leica microtome (Leica Microsystems Pty Ltd., NSW, AU) and stained with hematoxylin and eosin (H&E) or Tetrachrome. Spinal sides processed for PMMA histology were dehydrated through a series of increasing concentrations of ethanol and embedded in PMMA. A Leica SP1600 saw‐microtome was used to cut three sagittal sections of ~15 μm thickness from the medial, central, and lateral thirds of the fusion mass which were then stained with methylene blue and basic fuchsin.

Decalcified histology sections were used for the assessment of cell and tissue responses at the fusion site by investigators using high magnification light microscopy. Additionally, implant biocompatibility with respect to local effects was assessed by a certified veterinary pathologist using the semi‐quantitative scoring system of Annex E of ISO 10993‐6:2016 Biological Evaluation of Medical Devices—Part 6: Tests for Local Effects after Implantation with comparisons between the novel sCaP/Col I and the other clinically established CaP materials which served as controls for each of the respective manners of use as bone graft extenders or substitutes. From a single centrally cut section, local effects were scored across four high‐powered fields of view at the transverse process and four in the center of the fusion mass. A mean value for each animal was used in the final evaluation.

Undecalcified histology sections were used for quantitative histomorphometry using low magnification images. A region of interest (ROI) was selected by an experienced and blinded investigator using a polygon technique. CaP graft material and bone tissues (i.e., bone and marrow) were identified by pixel color and morphology. Their respective areas were determined as a percentage of the ROI area. Due to original differences in fusion mass composition inherent to the use of materials as bone graft extenders or substitutes, the reported comparisons have been limited to those between CaP groups with like‐for‐like use and between the CaP groups and the autograft control group.

### 2.7 Statistics

Fusion rates were analyzed by Kruskal‐Wallis tests. Histomorphometry and mechanical range of motion data were analyzed by ANOVA with multiple comparisons Games‐Howell post hoc tests when appropriate. Statistical analyses were conducted in IBM SPSS version 24 with the alpha value set at 0.05.

## 3 Results

### 3.1 Material Characterization

**FIGURE 1:**
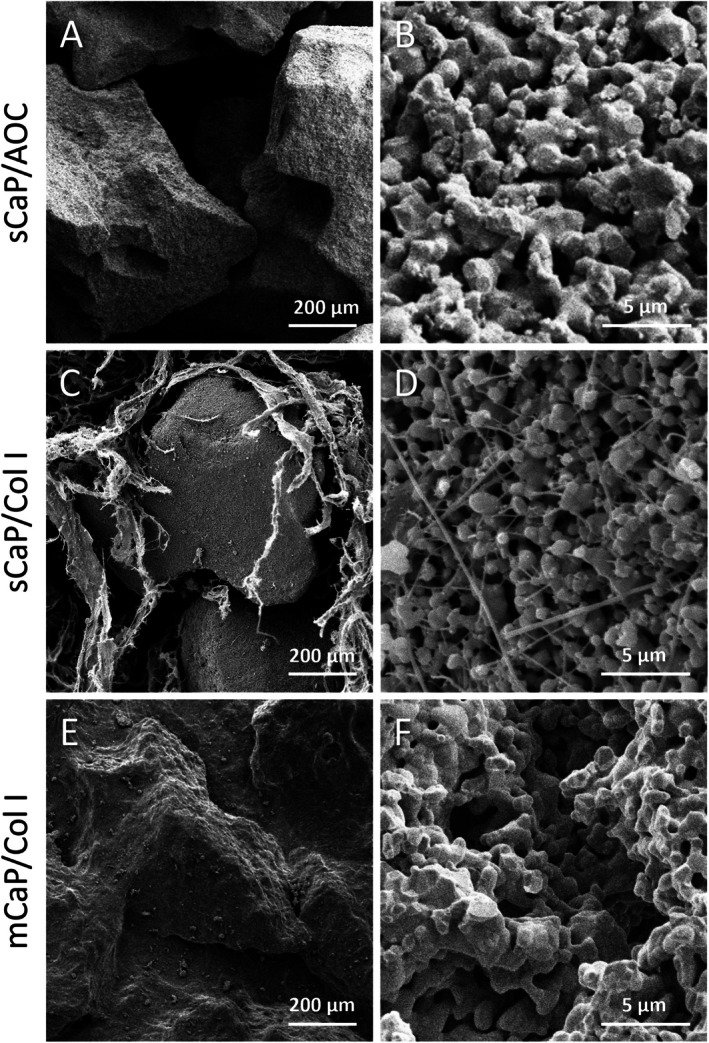
Graft microstructure represented by scanning electron micrographs. sCaP granules (A, B) isolated from sCaP/AOC graft and (C, D) embedded within the collagen matrix of sCaP/Col I graft. (E, F) mCaP granules embedded within the collagen matrix of mCaP/Col I graft.

**FIGURE 2:**
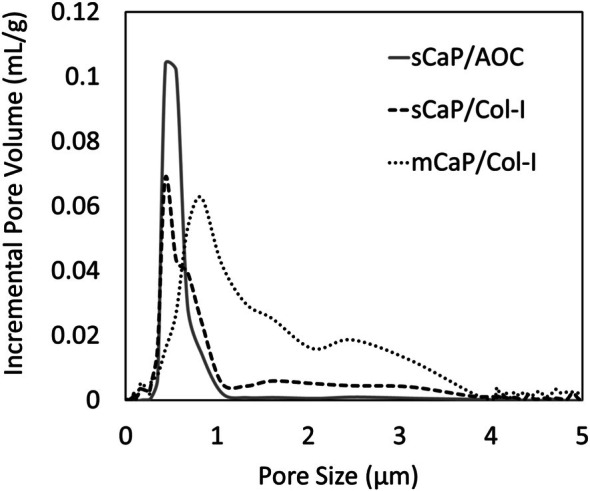
Microporosimetry analysis shows microstructural differences between sCaP and mCaP graft materials.

The two clinically available sCaP graft materials tested in this study contained identically manufactured ceramic granules but differed in resorbable binder compositions: one with bovine collagen type I and the other with an alkylene oxide copolymer (AOC) (Table 1). The sCaP in these materials was characterized by a tightly controlled distribution of submicron‐sized pores (~0.3–1.1 μm) which was apparent from the uniform sCaP surface structure observed by SEM (Figure 1) and from quantitative microporosimetry of pore size distribution (Figure 2). For pores less than 10 μm in diameter, > 96% of the pore volume in the sCaP comprised submicron (< 1 μm) sized pores. In contrast, the clinically available mCaP/Col I test article was observed to have ceramic granules with a more variable surface structure. The mCaP pores spanned a wider range of sizes (~0.3–3.5 μm), and only ~30% of their volume comprised submicron pores. Differences observed between graft materials by SEM were not just limited to the CaP structure but also included differences in the morphology of collagen binders. The collagen in the sCaP/Col I was more fibrous compared to the more sheet‐like structure observed in mCaP/Col I.

### 3.2 Health Observations

Postoperative radiographs were used to confirm the proper placement of graft materials across the transverse processes at the correctly operated level. No adverse effects were noted with any of the graft materials. No abnormalities were detected in the blood work (biochemistry and hematology) preoperatively or at the time of euthanasia. One subject in the 6‐week sCaP/AOC with autograft group was removed from analysis due to a respiratory issue unrelated to the material. Upon necropsy, a lung abscess was found. The subject was not replaced.

### 3.3 Radiographic Outcomes

**FIGURE 3:**
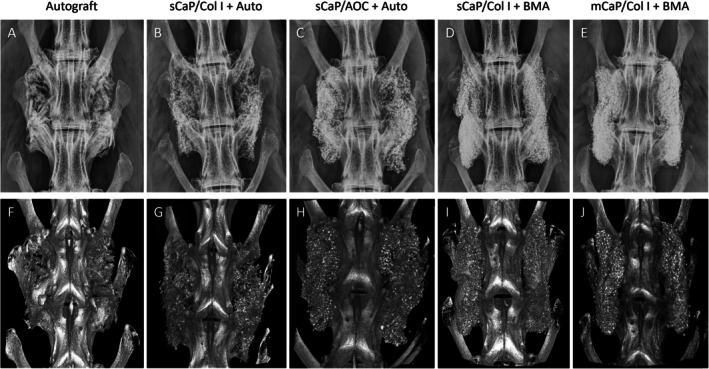
(A–E) Faxitron radiographs and (F–J) corresponding micro‐CT models of spinal fusions representative of each treatment group at 12 weeks.

By radiographic assessments, all groups demonstrated progressive new bone formation, remodeling, and partial resorption of graft materials within the fusion masses from 6 to 12 weeks (Figure 3). Based on the evaluation of multiplanar serial sections and the 3D models of micro‐CT, bilateral fusion rates generally improved with time (Table 3). Few radiographic fusions were observed in any group at the earliest time point of 6 weeks, and fusion rates were not significantly different at this time (*p* = 0.661). Notably, sCaP/Col I with autograft or BMA had significantly higher fusion rates by micro‐CT at 9 weeks compared to mCaP/Col I with BMA (*p* = 0.008 and *p* = 0.032, respectively). At 12 weeks, fusion rates were again not significantly different among groups (*p* = 0.181). The overall incidence of radiographic fusion at 9 weeks and beyond demonstrated that the novel sCaP/Col I graft with autograft (85%) performed at least as well as autograft (46%) and sCaP/AOC with autograft (69%). Likewise, sCaP/Col I with BMA (54%) also performed at least as well as autograft (46%) and mCaP/Col I with BMA (15%).

**TABLE 3:** Bilateral fusion incidence assessed by manual palpation and micro‐CT (number fused/number assessed).

Test group	Fusion by manual palpation			Fusion by micro‐CT		
	6 weeks	9 weeks	12 weeks	6 weeks	9 weeks	12 weeks
Autograft	1/5 (20%)	4/5 (80%)	6/8 (75%)	1/5 (20%)	2/5 (40%)	4/8 (50%)
sCaP/Col I + Auto	0/5 (0%)	5/5 (100%)	6/8 (75%)	0/5 (0%)	5/5 (100%)*	6/8 (75%)
sCaP/AOC + Auto	1/4 (25%)	2/5 (40%)	6/8 (75%)	1/4 (25%)	3/5 (60%)	6/8 (75%)
sCaP/Col I + BMA	1/5 (20%)	4/5 (80%)	6/8 (75%)	1/5 (20%)	4/5 (80%)**	3/8 (38%)
mCaP/Col I + BMA	1/5 (20%)	2/5 (40%)	4/8 (50%)	0/5 (0%)	0/5 (0%)*,**	2/8 (25%)

* *p* = 0.008 versus other indicated treatment.

** *p* = 0.032 versus other indicated treatment.

### 3.4 Mechanical Testing Outcomes

Fusion rates assessed by manual palpation progressed with time, and no statistical differences were observed between groups at each time point of 6, 9, and 12 weeks (*p* = 0.867, *p* = 0.174, and *p* = 0.762, respectively; Table 3). Similar to the radiographic fusion findings, few stable fusion masses were found among groups at the early time point of 6 weeks. The overall incidence of radiographic fusion at 9 weeks and beyond demonstrated that the novel sCaP/Col I graft with autograft (85%) and with BMA (77%) performed at least as well as all other groups: autograft (77%), sCaP/AOC with autograft (62%), and mCaP/Col I with BMA (46%).

Robotic range of motion testing of spinal segments at 12 weeks supported the manual palpation outcomes. Compared to nonoperated spinal segments, the mean range of motion was reduced in all groups (range: −24% to −59% for FE; −25% to −70% for LB; and −11% to −47% for AR) reaching significance in all groups for FE (*p* < 0.05) and in the autograft, sCaP/Col I with autograft, and sCaP/AOC with autograft groups in LB (*p* < 0.05).

### 3.5 Histology Outcomes

**FIGURE 4:**
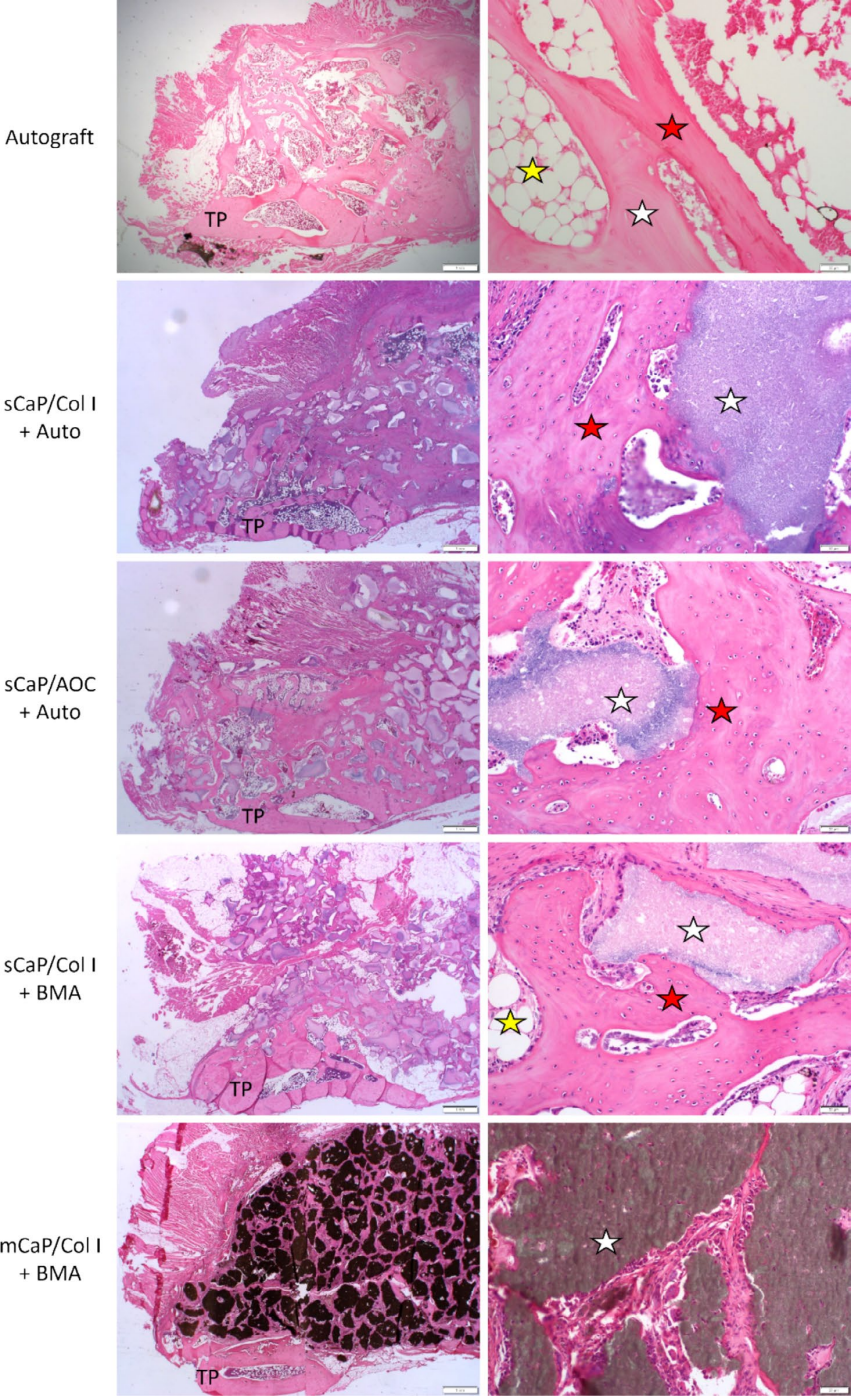
Histology of fusion masses at 12 weeks shows tissue formation within the residual graft material adjacent to transverse processes (TP). Scale bars: Left = 1 mm; right = 50 μm. Red stars = new bone. Yellow stars = new marrow. White stars = residual graft material (autograft or CaP).

Histology further supported the observed progression of fusion with time in all groups in a manner consistent with radiographic and mechanical results. New bone and marrow formation and loosely organized fibrous tissue were observed throughout the residual bone graft materials (Figure 4). The earliest and most active bone formation was generally found adjacent to the transverse processes and was more apparent in the intertransverse region at later time points. In all CaP graft material groups, new bone was observed to be forming directly on the surface of residual CaP granules and spanning between granules. Autograft and residual CaP granules were undergoing the expected resorption process, and occasional multinucleated cells were observed on the material surfaces.

Semi‐quantitative assessment of the histology sections for local effects per ISO 10993‐6 revealed that the novel sCaP/Col I graft material performed in a nonreactive manner relative to the other clinically established CaP grafts. No adverse biological effects were noted with any of the graft materials.

**FIGURE 5:**
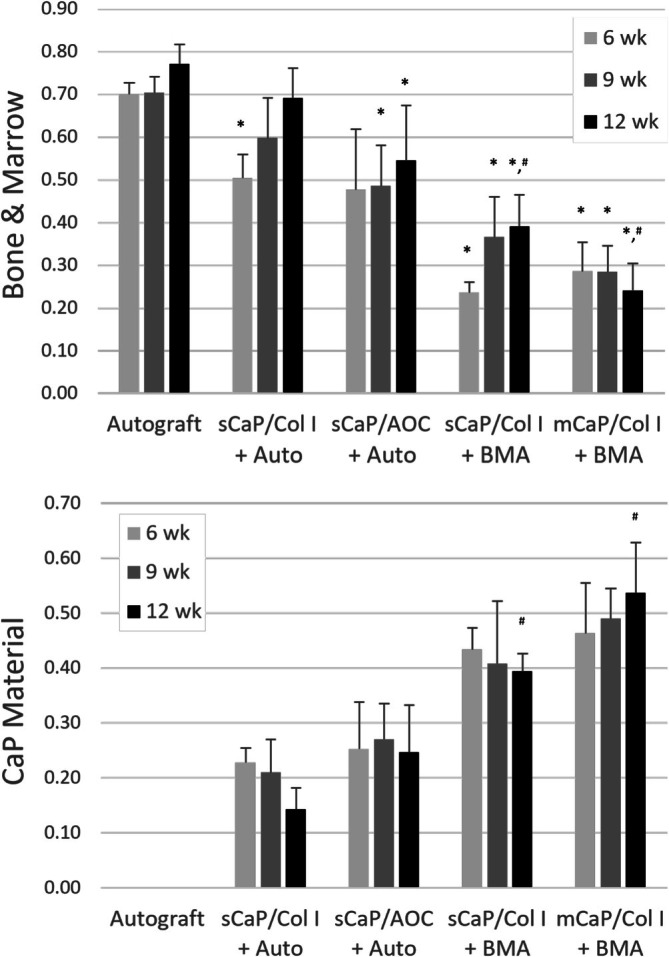
Histomorphology analysis of fusion mass composition shows bone and marrow content (upper panel) and CaP graft material content (lower panel) normalized by total fusion mass area ROI (mean + SD; *p < 0.05 vs. autograft at same time point; ^#^ *p* < 0.01 vs. other indicated treatment at the same time point).

Histomorphometry was used to quantify bone tissues, inclusive of new bone, marrow, and residual implanted autograft when present, and residual CaP graft material as a fraction of the fusion mass ROI. All groups, except sCaP/Col I with autograft, had a lower bone and marrow tissue content compared to autograft at 9 and 12 weeks (*p* < 0.05; Figure 5). If alternatively normalized to the area available for tissue formation (i.e., total ROI area minus CaP material area), only the bone and marrow content of the two CaP groups with BMA remained significantly lower than autograft at the final time point of 12 weeks (*p* < 0.05). Additionally, the sCaP/Col I with BMA group showed greater bone and marrow content at 12 weeks compared to mCaP/Col I with BMA (*p* = 0.006). When assessing residual CaP material, the determination of specific graft resorption rates was not practical due to competing effects of resorption and graft consolidation, i.e., densification as binders were remodeled and resorbed. By the final 12‐week time point, sCaP/Col I with BMA showed significantly less residual CaP material than mCaP/Col I with BMA (*p* = 0.008; Figure 5).

## 4 Discussion

The current study sought to compare the performance of 3 clinically available CaP‐based bone graft materials, including a novel sCaP/Col I graft previously uncharacterized in the literature, and iliac crest autograft in a standardized rabbit PLF model. All materials supported a progression of fusion through the 12‐week postoperative study duration, with increasing radiographic evidence of bridging bone, improvements in mechanical stability of the spinal segments, and histological observations of new bone and marrow formation. However, not all materials performed identically in all measures. These observed differences are likely indicators of the effects of differing material formulations and structures on the biological processes within the fusion sites, which may have implications for their clinical use.

Iliac crest autograft was used as a clinically relevant comparative material and model control group in this study. The outcomes for the autograft control group are highly consistent with the authors' prior published experience with this model, e.g., the 75% (6/8) fusion rate by manual palpation at 12 weeks in the current study is comparable to the overall rate of 76.9% (80/104) observed in a large series of studies [17]. Moreover, the similar radiographic fusion rates between the clinically established sCaP/AOC graft material and autograft echo those measured in high‐quality randomized controlled trials for human spinal fusion which have found sCap/AOC to be non‐inferior to autograft even when used as a bone graft substitute [9, 10, 11]. These results underscore the validity and translational relevance of this rabbit PLF model.

The novel formulation of sCaP with collagen type I also performed as well as iliac crest autograft and sCaP/AOC, which serve as comparative benchmarks for safety and effectiveness for the new material. Notably, both sCaP formulations supported similar achievement of radiographic and mechanical fusion in the majority of spinal segments at 9 weeks or later. Furthermore, fusion outcomes with sCaP/Col I were similar when used as either a bone graft substitute or bone graft extender, two methods of use that may be particularly relevant to varied clinical scenarios where autograft bone may be limited in quality or quantity.

Prior preclinical testing has found that the composition of the binder component of bone graft putties and gels containing sCaP can influence the amount of bone formation within the materials, even abolishing it within some formulations [22, 23]. Therefore, careful attention must be paid to the selection and processing of binders during graft material development and manufacturing to ensure the final formulation performs as intended for bony healing. The AOC binder within sCaP/AOC has been well documented to resorb quickly from bone defects without impairing osteogenesis or eliciting adverse biological reactions [24, 25, 26]. Likewise, collagen has an extensive history of use as a binder in CaP‐based bone grafting materials, though the processed and purified bovine collagen type I used in sCaP/Col I was previously untested [1]. Despite their differing binder compositions, the two sCaP‐based grafts performed in a similar manner in the rabbit PLF model. Not only did these two graft materials achieve similar radiographic and mechanical fusion outcomes, but they also demonstrated comparable bone and marrow formation and biocompatibility. Thus, the bovine collagen type I binder neither inhibited bone formation nor generated greater inflammatory or immune reactions compared to AOC. Given the similar performance between these formulations of sCaP‐based grafts, distinctions in their use may be primarily influenced by individual clinician preferences for handling and absorbency properties.

When compared to an earlier generation graft material consisting of mCaP/Col I, the novel composition of sCaP/Col I demonstrated earlier fusion, with radiographic fusion rates significantly higher at 9 weeks (i.e., 80%–100% vs. 0%). Significantly more bone tissue formation was also measured by histomorphometry at 12 weeks with sCaP/Col I compared to mCaP/Col I. In previous research, CaP granules comparable to those used in the manufacture of these two graft materials have shown different intramuscular bone‐forming potential, with sCaP driving robust bone formation while mCaP formed none [8]. This divergent ectopic bone‐forming potential may be responsible, at least in part, for the performance differences observed in the rabbit PLF model with the clinically available forms of each ceramic. These results offer further evidence to a robust body of literature that optimized CaP microstructure can be a key instructor of osteogenesis and bony healing in preclinical testing [2, 3, 4, 5, 6]. While these results anticipate that sCaP grafts may provide meaningfully improved outcomes in clinical applications of spinal fusion compared to earlier generation grafts without optimized structures, further research using well‐controlled clinical study designs would be informative.

The rabbit PLF model is a challenging test for ceramic bone graft materials given the lack of initial stability and limited contact between graft materials and decorticated bone surfaces. Nevertheless, the interpretation of results from this study is subject to limitations of the choice of the model, the time points, and employed methods. For example, the lack of histology at the time of implantation limits our understanding of absolute changes in the composition within the fusion masses from a baseline value. The performance of the tested bone grafting materials may additionally differ in other translational animal models or clinical applications where the bone graft implantation environment varies from the rabbit PLF model.

Lastly, the tested CaP bone grafting materials have several confounding formulation variables. This study did not explicitly seek to test the effect of a single well‐controlled variable (e.g., CaP surface topography, β‐TCP:HA ratio, etc.) on fusion performance since many other studies have reported on these effects to guide biomaterials development. Rather, the focus of this study was on the comparison of graft material formulations currently available for clinical use to provide translatable findings. In particular, this study sought to characterize the performance of a novel sCaP/Col I formulation as both an autograft extender when combined with iliac crest bone graft and as an autograft substitute when combined with BMA. While these methods of use are common in the clinical setting and consistent with the applicable graft material instructions, alternative preparations of the graft material may yield different results.

## 5 Conclusion

This study provides comparative performance data of clinically available submicron‐structured and microstructured CaP‐based bone grafting materials in a translational model of posterolateral spine fusion. The results offer evidence that differences in composition and structure, including the optimization of CaP surface features in the submicron range, can influence the progression of bony healing and ultimately the successful achievement of fusion. Other differences, such as the substitution of the binder material in sCaP formulations, had negligible effects on fusion. These findings in combination with existing preclinical and clinical evidence may help to inform clinicians on the use of these graft materials and further guide the development of more advanced bone grafting materials in the future.

## Conflicts of Interest

C.T.D. and G.M.W. are employed by and hold stock or options of Globus Medical. The other authors declare no conflicts of interest.
